# The Ability of Triple Antibiotic Paste and Calcium Hydroxide in Disinfection of Dentinal Tubules

**Published:** 2014-03-08

**Authors:** Alireza Adl, Sabie Hamedi, Mahdi Sedigh Shams, Mohamad Motamedifar, Fereshte Sobhnamayan

**Affiliations:** a *Department of Endodontics, Dental School, Shiraz University of Medical Sciences, Shiraz, Iran*

**Keywords:** Antibiotic, Bacterial Infection, Calcium Hydroxide, *Enterococcus faecalis*, Root Canal Medicaments, Triple Antibiotic Paste

## Abstract

**Introduction:** The purpose of this *in vitro* study was to compare the ability of triple antibiotic paste (TAP) to calcium hydroxide (CH) in disinfecting dentinal tubules. **Material and Methods:** Sixty root blocks were obtained from extracted single-rooted human teeth. The root canals were enlarged with Gates-Glidden drills up to size 3 and were contaminated with *Enterococcus. faecalis* (*E. faecalis*), and then left for 21 days. The contaminated blocks were treated with saline (as negative control), CH or TAP. Dentin debris was obtained at the end of first and 7th days, using Gates-Glidden drills sizes 4 and 5 from two different depths of 100 and 200 µm. The vital bacterial load was assessed by counting the number of colony forming units (CFUs). The data was analyzed with the Kruskal-Wallis H and Dunn Post-Hoc tests. The Wilcoxon Signed Ranks test was used to check for differences in bacterial growth at both depths (*P*<0.05). **Results:** In comparison with CH, the TAP significantly decreased the number of CFUs in both depths and time intervals (*P*<0.001), while the CH group showed a moderate antibacterial effect. **Conclusion:** TAP is more effective in disinfecting the canal against *E. faecalis* compared to CH.

## Introduction

Microorganisms have an essential role in the onset and maintenance of pulp and periapical diseases; hence their elimination from the root canal system during endodontic treatment is very important [1]. Instrumentation of the canals, using antimicrobial irrigants reduces the bacterial population effectively; however, some bacteria may survive in the lateral and accessory root canals, isthmi, and apical deltas [2, 3].

Microbiological researches have shown that *Enterococcus faecalis (E. faecalis)*, a gram-positive facultative anaerobe, is more likely to be isolated from failed cases of root canal therapy [4]. This microorganism can deeply invade dentinal tubules and tolerate periods of starvation and extremities of pH [5]. It is generally believed that inter-appointment dressing of the root canal with an antimicrobial medicament helps in the elimination of residual microorganisms, thereby providing a condition to enhance the success of endodontic treatment [6]. Calcium hydroxide (CH) is the most common intracanal medicament that has the ability to kill the bacteria because of its high alkalinity. The high pH of CH changes the biologic properties of the lipopolysacharide component in the cell wall of gram negative species and inactivates the mechanisms of membrane transportation, which leads to bacterial cell death [7]. However, *E. faecalis* is rather resistant to CH because of its ability to penetrate deeper layers of dentinal tubules, where its pH is neutralized by the buffering action of dentin [8, 9]. Furthermore, CH reduces the flexural strength of dentin [10] and its efficacy in reducing the number of bacteria, even after prolonged contact with root canal walls, has been questioned [10]. Considering the shortcomings of CH, finding an alternative intracanal medicament would be beneficial.

The triple antibiotic paste (TAP), is a mixture of metronidazole, ciprofloxacin, and minocycline which is used as an intracanal medicament for disinfection of immature necrotic teeth, during regenerative endodontic procedures [11]. The effectiveness of this mixture against the common endodontic pathogens has been proved in several studies [12-14]. A recent *in vitro* study showed that compared to CH, the TAP is highly effective against *E. faecalis* [15]. Another study on teeth contaminated with *E. faecalis*, reported that TAP can reduce the colony count more effectively than CH [16]. However, no studies exist on the ability of TAP to penetrate and disinfect the dentinal tubules. Hence, the aim of the present *in vitro* study was to evaluate the disinfection of dentinal tubules contaminated with *E. faecalis *by the TAP compared to CH, as intracanal medicaments.

## Methods and Materials

The model previously described by Haapasalo and Ørstavik [17], was modified for this study, and 60 extracted single-rooted human teeth with straight root canals were selected.

***2.1. Preparation of the blocks***

Slices with 6 mm length from the middle third of each root were obtained by cutting the coronal and apical parts of the roots. To standardize the internal diameter of the blocks, Gates-Glidden drills sizes 1, 2, and 3 (Mani Inc, Takanezawa, Japan) were used to enlarge the canals. To remove organic and inorganic debris, the blocks were submerged in an ultrasonic bath of 17% ethylenediaminetetraacetic acid (EDTA, Asia Chemi Teb Co., Tehran, Iran) followed by 2.5% sodium hypochlorite, for 5 min each. Finally, the samples were immersed in an ultrasonic bath of distilled water for another 5 min in order to remove any trace of the used chemicals and then sterilized in an autoclave at 121° C for 30 min. A second cycle of sterilization was done while the root blocks were immersed in 1 mL of tryptic soy broth (TSB, Oxoid Limited, Basingstoke, Hampshire, England) in individual microcentrifuge tubes, to allow better penetration of TSB into dentinal tubules.

***2.2. Contamination of the blocks***

Pure culture of *E. faecalis* (ATCC 11700) was used as the test organism in this study. For contamination of the specimens, each block was transferred to a pre-sterilized microcentrifuge tube containing 1 mL of the TS broth and then 50 µL of an inoculum of *E. faecalis*, equivalent to 0.5 McFarland standard (1.5×10 CUF/mL), was added to each tube. Every two days, the blocks were transferred to fresh TSB containing *E. faecalis*, during a period of three weeks. All the procedures were done under laminar flow hood (Class I, Jal Tajhiz, Iran).

***2.3. Antimicrobial assessment***

At the end of the incubation period, the blocks were removed from the broth and irrigated with 5 mL of sterile saline. The samples were then assigned to the following groups (*n*=20): group 1, saline (negative control); group 2, calcium hydroxide (CH); group 3, triple antibiotic paste (TAP). CH powder (Golchay, Tehran, Iran) was mixed with sterile saline to obtain a paste-like consistency. To prepare the TAP, equal amounts (50 µg) of pure metronidazole, ciprofloxacin, and minocycline were mixed with sterile saline to obtain a paste-like consistency [18]. The pastes (groups 2 and 3) were carried into the canals using lentulo spirals (Dyna, Bourges, France). After removal of the excess medicament, coronal and apical orifices were sealed with paraffin wax. The specimens were then incubated at 37° C and 100% humidity.

Antimicrobial assessment was performed at the end of days 1 and 7, with 10 blocks from each group for each time interval. At the end of each time interval, the paraffin wax was removed, and the root canals were irrigated with 10 mL of sterile saline and then dried with sterile paper points (Ariadent, Tehran, Iran). Dentine debris was obtained using Gates-Glidden drills sizes 4 and 5 from the depths of 100 and 200 µm, under laminar flow hood. The debris were collected in microcentrifuge tubes containing 1 mL of sterile TSB and incubated in an anaerobic environment at 37° C for 24 h. After the incubation period, the content of each microcentrifuge tube was serially diluted; 100 mL of the broth in 900 mL of sterile broth for 3 times. At the end, 100 mL of this diluted sample was plated on TSB agar and incubated for 48 h. Colonies were counted, and readings were tabulated.

***2.4. Statistical analysis***

The data were statistically analyzed with the Kruskal-Wallis H and Dunn Post-Hoc tests to assess the differences in antibacterial efficacy between groups (*P*<0.05). The Wilcoxon Signed Ranks test was used to check for differences in bacterial growth in both depths (*P*<0.05).

## Results

The results are presented in Table 1. Infection of the blocks was confirmed as the saline group (negative control) showed heavy colonization of dentinal tubules with *E. faecalis. * The TAP was the most effective medicament against *E. faecalis*, as it showed significant differences with either saline and CH in both time intervals and depths. The CH group showed a moderate antibacterial effect as its difference with control group was significant in depth of 100 µm in the day seven. For all groups, the number of bacteria was less in depth of 200 µm than 100 µm. The difference between the two depths, in day 1 was significant just in saline group, while a significant difference was noticed in day 7, in all groups.

## Discussion

Various *in vitro* and *in vivo* models are proposed for the evaluation of antimicrobial efficacy of intracanal medicaments. The *in vitro* model used in this study was modified from the technique proposed by Haapasalo and Ørstavik [17]. Because of the remarkable differences in lumen size between the canals of bovine and human teeth, human permanent teeth were used in this study to simulate the clinical conditions. Furthermore, the model used in this study allowed assessment of the antimicrobial efficacy of tested medicaments on bacterial biofilms inside the dentinal tubules instead of planktonic microorganisms suspended at the lumen of the root canals [19, 20]. *E faecalis* was chosen for this study because of its association with cases of refractory disease after endodontic treatment [21-23] and its resistance to antibacterial substances [24, 25]. Although there are some questions regarding the correlation between endodontic failures and *E. faecalis* [26], many recent studies have used this microorganism to evaluate the effect of different antibacterial agents/techniques [27-29].

**Table1:** Median of colony counts for different intracanal medicaments at 100 and 200 µm depths at different time intervals

**Groups**	**Median (Interquartile Range) Colony Count(10** ^4^ **)**					
	**First day **			**Seventh day**		
	100 µm	200 µm	*P-*value b	100 µm	200 µm	*P-*value b
**Saline**	328.79 (229.29)	226.77 (98.93)	0.022	579.62 (417.76)	273.45 (216.71)	0.005
**Calcium hydroxide**	257.95 (217.59)	186.87 (199.88)	0.139	379.81 (281.33)	249.65 (175.27)	0.005
**Triple antibiotic paste**	0.0100 (1.36) b	0.0100 (1.12) b	0.236	0.0100 (1.16)	46.82 (60.97)	0.005
***P-*** **value ** a	<0.001	<0.001		<0.001	<0.001		

a *. Using Kruskal–Wallis H test;*

b *. Using Wilcoxon signed ranks test*

Based on the results of this study, *E. faecalis* was capable of penetrating up to 200 µm into the dentinal tubes, as debris obtained from depth of 100 µm and 200 µm of the saline group (negative control) showed bacterial recovery. This is in agreement with previous studies concerning the ability of *E. faecalis* to penetrate deeply into the dentinal tubules [30-32].

According to the present results, the TAP is more effective against *E. faecalis* compared to CH. This finding confirms a recent study by Madhubala *et al.*, in which propolis and TAP showed higher antibacterial effects than CH on *E. faecalis* [16]. It is worth mentioning that the aforementioned study did not examine the efficacy of penetration depth of tested medicaments at various depths of dentinal tubules; however, the present study showed that the TAP is able to penetrate the dentinal tubules up to 200 µm and reduces the bacterial count. Another interesting finding of this study was that the TAP can substantially reduce the number of bacteria even after one day. This is in agreement with a study by Sato *et al*. in which no bacteria were recovered from the infected dentin 24 h after application of the TAP, except for one case in which a few bacteria were recovered [14]. It is noteworthy to mention that Sato *et al*. dealt with a mixture of bacteria involved in endodontic infection contrary to the single-infection model used in the present study.

Because of complexity of the root canal infection, a combination of antibiotics may be needed to address the diverse flora encountered in root canal infections. A combination of antibiotics might also decrease the likelihood of the development of resistant bacterial strains [33]; however, one study showed that minocycline is the most effective component of the TAP against *E. faecalis* [15]. Minocycline is a semisynthetic derivative of tetracycline with a similar spectrum of activity against both gram positive and gram negative microorganisms. In the present study, the bactericidal effect of CH against *E. faecalis* was lower compared to the TAP (Table 1). This observation is in agreement with the growing concern about the limited antibacterial efficacy of the TAP against some microorganisms commonly found in infected root canals [34-36]. The insufficient antibacterial efficacy of CH has been attributed to the buffering action of dentin [9]. In contrast, there are also some reports confirming the effectiveness of CH against *E. faecalis* [37, 38]. Although the results of this study confirmed the bactericidal efficacy of the TAP against *E. faecalis*, clinical studies are necessary to illustrate its antimicrobial efficacy as an intracanal medicament, especially in non-surgical retreatment of endodontic failures.

## Conclusion

Under the limitations of this study, TAP is more effective against *E. faecalis* in comparison with CH.
